# Machine learning approach to gait analysis for Parkinson’s disease detection and severity classification

**DOI:** 10.3389/frobt.2025.1623529

**Published:** 2025-12-10

**Authors:** Rohit Mittal, Nikunj Agarwal, Manan Dubey, Vibhakar Pathak, Praveen Shukla, Geeta Rani, Eugenio Vocaturo, Ester Zumpano

**Affiliations:** 1 Department of IoT and Intelligent Systems, Manipal University Jaipur, Jaipur, India; 2 Department of Artificial Intelligence and Machine Learning, Manipal University Jaipur, Jaipur, India; 3 Department of Information Technology, Arya College of Engineering and I.T., Jaipur, India; 4 Department of Computer Engineering, Modeling, Electronics and Systems, University of Calabria, Cosenza, Italy; 5 National Research Council, Institute of Nanotechnology (CNR-NANOTEC) Ponte P. Bucci, Cosenza, Italy

**Keywords:** Industry 4.0 technologies, non-communicable diseases (ncds) healthcare, gait, machine learning, Parkinson, affordable

## Abstract

Parkinson’s Disease is a progressively advancing neurological condition. Its severity is evaluated by utilizing the Hoehn and Yahr staging scale. Such assessments may be inconsistent, are more time-consuming, and expensive for patients. To address these shortcomings, this article introduces a machine learning-based gait classification system to assist doctors in identifying the stages of Parkinson’s disease. This study utilizes two open-access benchmark datasets from PhysioNet and Figshare to assess ground reaction force collected from patients diagnosed with Parkinson’s Disease. This study presents experiments conducted using machine learning algorithms namely Decision Tree, Random Forest, Extreme Gradient Boost, and Light Gradient Boosting Machine classification algorithms to predict severity of Parkinson’s Disease. Among all the four algorithms, Light Gradient Boosting Machine classification algorithm have proven its supremacy. It gave an accuracy of 98.25%, Precision of 98.35%, Recall of 98.25%, and F1 Score of 98% for dataset 1. The performance of the algorithm slightly declines on dataset 2. It reports accuracy of 85%, Precision of 95%, Recall of 85% and F1 Score of 89% for dataset 2. Furthermore, this study used Explainable Artificial Intelligence to display the LightGBM classifier’s classification pathways for Parkinson’s disease severity prediction using Hoehn and Yahr staging on the scale from 0 to 5. This is helps the health experts in decision making. This work provides automated assistance to doctors for the rapid screening of Parkinson’s disease patients based on disease severity. This work leaves a scope for integrating wearable sensors and developing real-time monitoring system for screening of Parkinson’s Disease patients.

## 1 Introduction

Neurodegenerative disorders (NDDs) arise due to progressive death of neural cells (Balakrishnan et al., 2024; Lilienfeld and Perl, 1993). The most common NDDs include Alzheimer’s Disease (AD), Parkinson’s Disease (PD), Huntington’s Disease (HD), and Amyotrophic Lateral Sclerosis (ALS). As reported in Veeraragavan et al. (2020), more than 10 million people are suffering from PD. NDDs cause motor, cognitive, and autonomic dysfunction of nervous system. This may lead to disability and mortality (Mujib et al., 2025; Zeng et al., 2016). Early diagnosis of these disorders is the only measure for effective treatment. Present diagnosis of these disorders is primarily done based on the symptoms such as shaking, slowness, and impaired mobility (Jankovic, 2008). These symptoms may also appear due to other disorders, such as stroke and essential tremors. Also, the symptoms become more prominent at the severe stage of these diseases. Thus, an alternative method of diagnosis is highly required. Gait disturbance is one of the early indicators of PD in approximately 80% of patients (Huo et al., 2025). It includes rhythmic alternating movement of the trunk and limbs while walking. Such impairments increase the risk of falls, leading to fractures and injuries. Clinical observation and motion capture systems for Gait disturbances are subjective and time-consuming. These difficulties motivated the researchers to use wearable sensors and machine learning (ML) algorithms for gait analysis driven by information and communication technology (ICT). ML algorithms have already proven their significance in disease diagnosis and patient care (Agarwal et al., 2024; Pradhan et al., 2022; Kundu et al., 2020). But, these algorithms work as a black-box and hard to interpret the decision making process. This reduces clinicians’ confidence and patients’ willingness to accept it. Explainable Artificial Intelligence (XAI) frameworks can overcome this limitation and improves transparency in decision making. This study takes advantage and proposes a framework that focuses on applying ML algorithms including LightGBM, DT, RF, and XGBoost for PD severity prediction followed by result validation through XAI.

This study aims to improve the accuracy and reliability of PD detection using gait analysis. Initially, it focuses on identifying distinguishing features between PD patients and healthy individuals, followed by applying a deep learning model for early detection of PD using PhysioNet Gait (Dataset 1) and Figshare (Dataset 2) datasets (Balakrishnan et al., 2024; Boari, 2022). The major contributions of this research are.

1. To effectively handle null values and compute the Center of Pressure (COP) from foot sensor data to assess instability in Parkinson’s patients.
2. To mitigate the problem of class imbalance using the SMOTE technique.
3. Item Comparative analysis of ML models to select the base model for PD detection and severity prediction.
4. To improve accuracy of Early PD detection, and severity prediction by fine-tuning the base model “LightGBM classifier”.
5. To employ Explainable AI methods for enhancing transparency and reliability of “LightGBM classifier” applied for early Parkinson’s Disease detection.

## 2 Literature review

Various studies conducted in the literature (Huo et al., 2025) investigated how participants suffering from PD used their brains and how dual activities affected their gait. The researchers in Faiem et al. (2024) proposed a deep learning-based multimodal approach that integrates gait and speech data to bring diversity in the dataset. The approach reported an accuracy of 97.3%. Next, the authors Marin-Lopez et al. (2024) focused on multiscale entropy analysis of gait data, showing its potential in assessing variability in locomotion patterns. Researchers rely on multiscale entropy analysis of gait data, which may overlook dynamic gait characteristics (Raghuvanshi et al., 2024) for real-time monitoring of gait abnormalities in PD patients. The aforementioned techniques were applied to gait dataset comprising healthy controls and Parkinson’s patients. Thus, it fails to diagnose PD at an early stage. To overcome this challenge, the researchers (Zhao et al., 2022) developed a hybrid spatio-temporal model that has the potential to diagnose PD at different stages based on their gait data. The model gave an accuracy of 95.02%. Similarly, the authors Senturk (2020) applied ML techniques to analyze voice signal features for differentiating between healthy individuals and PD patients. Their technique reported the highest accuracy of 93.84% using SVM. Similarly, the authors Khoury et al. (2019) applied KNN, DT, RF, NB, and SVM models to analyze gait characteristics. They reported the highest accuracy of 92.86%. The authors in reference Aşuroğlu et al. (2018) applied Locally Weighted Random Forest (LWRF) on gait signals collected from PD patients. They achieved a classification accuracy of 99% on H&Y scale. Such models had achieved high accuracy in classifying PD gait abnormalities, even in preclinical stages. The performance of all the above-discussed models is dependent on the quality and quantity of the training data. Thus, the availability of limited diversity in the dataset limits generalizability and causes the problem of overfitting in these models. Also, none of the methods discussed above focuses on early-stage diagnosis of PD. The above discussion shows that various machine-learning models can be applied to the collected data for PD diagnosis. However, none of these worked on real-time data collection, analysis, and diagnosis. Therefore, there is a scope to include wearable and non-wearable sensors (Marsico and Mecca, 2019) for data collection.

The existing studies rely on open datasets, which do not capture the variability in clinical conditions. Further, wearable sensor-based approaches lack large-scale validation across diverse patient populations. This may cause potential sampling bias. Also, the challenges related to sensor calibration, and longitudinal monitoring of disease progression are overlooked. Thus, the applicability of existing studies is limited due to the absence of prospective validation. Our study addresses these shortcomings. The authors in this research effectively handle the null values and compute the COP from foot sensor data, offering a measure of instability in PD patients. Further, they apply Synthetic Minority Oversampling Technique (SMOTE) technique to overcome the limitation of class imbalance observed in PD datasets. This is important to improve H&Y stages and model generalization. In addition, Explainable AI techniques are integrated to bring transparency in decision-making mechanism of the ML model and to improve reliability on classification results. This may enhance clinical trust and applicability of this model in early PD detection.

## 3 Methodology

**FIGURE 1:**
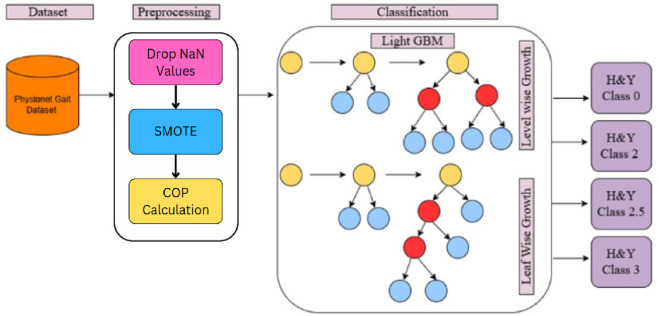
Flowchart of Parkinson’s Disease classification framework.

The proposed approach starts with importing the dataset into workspace, followed by a series of pre-processing steps to extract the necessary data. It includes dropping of Null values (NaN), reducing the impact of class imbalance using SMOTE. Next, it calculates the Center of Pressure (COP) through model training, and applies Decision Tree (DT), Random Forest (RF), XGBoost,and LightGBMLightGBM models for classification of Parkinson’s disease severity according to the H&Y scale. Figure 1 represents an overview of the classification framework.

### 3.1 Dataset 1

This study utilized the open-access benchmark dataset from PhysioNet to analyze Ground Reaction Force (GRF) data from PD patients (Hausdorff, 2008). The gait dataset, gathered at Tel-Aviv Sourasky Medical Center (Brand et al., 2022), contains assessments of PD severity using conventional clinical methods such as H&Y and Unified Parkinson’s Disease Rating Scale (UPDRS). The dataset includes features for early PD stages, and serves as a benchmark dataset for disease stage identification using statistical and kinematic analysis. The dataset details are demonstrated in Table 1.

**TABLE 1:** Dataset description.

Column number	Description	Details
1	Time	Time elapsed during walking trial
2–9	Left foot sensors	Vertical ground reaction force (VGRF) from 8 sensors under the left foot
10–17	Right foot sensors	Vertical ground reaction force (VGRF) from 8 sensors under the right foot
18	Total left force	Sum of 8 sensor readings under the left foot
19	Total right force	Sum of 8 sensor readings under the right foot

Further, gait cycles were analyzed to examine sequences of foot contacts during walking. Motor control is assessed by step length, cadence, and velocity. Such analysis is necessary for neurological illness monitoring. The dataset includes information collected by three study groups (Ga, Ju, and Si) (Balaji et al., 2021b). Each of them recruited healthy controls (CO) and PD patients for collecting and performing quantitative analysis of gait data. The PhysioNet Gait dataset also contains demographic information, including age, weight, and height, for participants with health problems. The dataset consists of 279 gait recordings from 73 healthy subjects including 40 men and 33 women. The mean age of patients is 63.7 years. Further, the dataset contains 93 idiopathic PD patients, including 58 men and 35 women. The mean age of patients is 66.3 years. The severity level of PD patients varies from mild, medium, and high. Table 2 shows demographic characteristics of both healthy controls and PD individuals.

**TABLE 2:** Demographic information of subjects across groups.

Group	Subjects		Age (Years)		Weight (Kg)		Height (m)
	M	F	M	F	M	F	
Healthy (0)	40	33	63.87 ± 42.16	63.47 ± 38.07	64.69 ± 25.57	79.10 ± 19.08	1.68 ± 0.08
Mild (2)	36	19	61.6 ± 19.01	65.3 ± 11.42	64.66 ± 17.14	78.67 ± 9.44	1.67 ± 0.06
Medium (2.5)	16	12	69.9 ± 10.39	68.6 ± 11.13	66.75 ± 8.53	79.9 ± 7.13	1.71 ± 0.07
High (3)	6	4	64.2 ± 11.58	75.0 ± 11.20	59.75 ± 6.29	67.2 ± 6.61	1.68 ± 0.06

**FIGURE 2:**
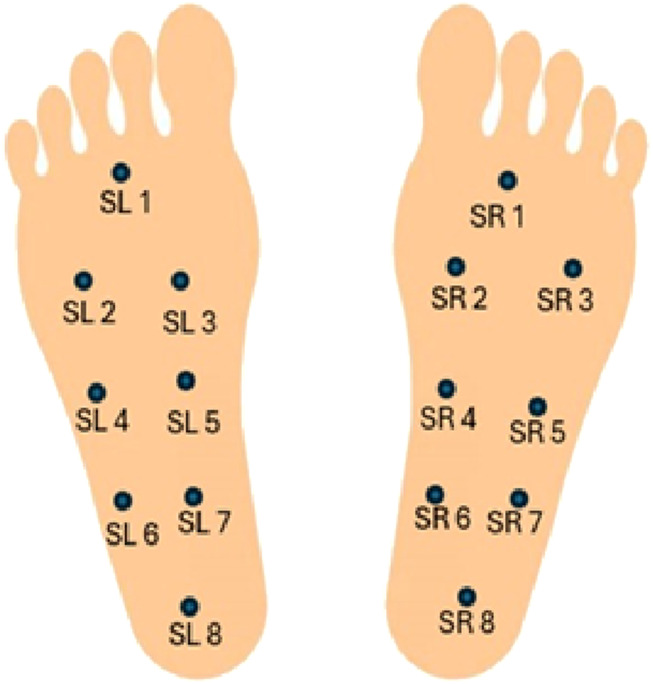
Visual representation of the VGRF sensor placement.

The data was collected using eight GRF sensors (SL1-SL8 and SR1-SR8) placed under each foot. The sensors were enabled to capture a detailed pressure distribution line with the experimental protocol. The coordinates of these sensors lie at the underfoot plane, when a person is standing with both legs parallel. Each participant was asked to walk at their self-selected pace on ground level for about 2 minutes. The vertical ground reaction force (VGRF), measured in Newtons, is generated when the foot made contact with the ground. Figure 2 provides a visual representation of the VGRF sensor placement.

The sensor outputs were recorded by an A/D converter at a sampling rate of 100 Hz. Each record consists of 19 parameters, where each row represents a set of these parameters. It includes the timestamp in seconds, 16 VGRF values measured in Newton corresponding to the 16 force sensors under both feet, and 2 total VGRF values.

### 3.2 Dataset 2

This study utilizes a publicly available dataset available at Boari (2022). The dataset represents gait biomechanics including full-body kinematics and kinetics made during overground walking by 26 healthy participants with idiopathic PD. The age of participants was between 44 and 81 years. Each participant comprised two walking tests, one in the ON-medication state, after a steady dose of L-DOPA and one in OFF-medication state, following a minimum of 12-h withdrawal. A 3D motion-capture system that comprises 44 reflective markers was used to measure variables related to kinematics, whereas variables related to kinetics were measured by a 3D over-ground walking test using embedded force plates. The data includes raw data in.c3d and processed data in.csv formats. It also contains comprehensive clinical metadata, such as the UPDRS scores, cognitive assessment (MoCA, Stroop, Trail Making A/B), balance and mobility (Mini-BESTest, FES-I), and demographics. This experiment was done at the Laboratory of Biomechanics and Motor Control of the Federal University of ABC, Brazil, and passed ethical analysis (protocol number 21948619.6.0000.5594). The data is freely distributed through Figshare.

### 3.3 Pre-processing

This section elaborates pre-processing mechanism followed to prepare the dataset.

#### 3.3.1 Handling class imbalance using SMOTE

Class imbalance is a major challenge observed in the dataset collected for this research. The data points with H and Y stages having mild symptoms were higher than advanced stages data points. This may lead to biased predictions and poor generalization of ML model. To mitigate the effects, the SMOTE algorithm is applied. It analyzes the class distribution, identifies minority classes, and interpolates between existing instances to generate synthetic samples for classes with lesser data points. This approach not only increases the volume of training data but also enhances the model’s ability to recognize subtle patterns associated with less common H and Y stages.

#### 3.3.2 Center of pressure (COP) calculation

COP feature represents the point where GRF is applied. COP is computed by determining the weighted average of the sensor readings. Each foot has eight sensors, which are evenly spaced along a predefined axis. The COP is calculated by adding the product of sensor activations and their spatial positions, normalized by the total force exerted by the foot. This provides both X-and Y-coordinates of the COP for the left and right feet. In case there is no contact of the foot with ground, then values of the coordinates are recorded as zero. Hence, its corresponding COP values are set to zero to prevent division by zero errors. COP for the left and right feet is calculated using Equations 1 and 2, respectively (Afzal et al., 2023; Alam et al., 2017). From the calculated COP coordinates, other features like mean COP, which reflects the average balance point during a gait cycle, and the COP variability, which quantifies the fluctuations in pressure distribution, are derived. Increased variability indicates the postural instability, a hallmark of advanced Parkinson’s disease. Each patient’s H&Y stage is mapped to their corresponding gait data using the unique subject identifier. By aligning the COP features with these labels, the dataset becomes suitable for supervised learning. By analyzing COP dynamics, it is possible to quantify motor impairments characteristic of PD. Left foot:

$${\text{COP}}_{\text{Left},x}=\frac{\sum_{i=1}^{N}F_{\text{Left},i}\times x_{i}}{\sum_{i=1}^{N}F_{\text{Left},i}},\;{\text{COP}}_{\text{Left},y}=\frac{\sum_{i=1}^{N}F_{\text{Left},i}\times y_{i}}{\sum_{i=1}^{N}F_{\text{Left},i}}$$ (1)

Right Foot:

$${\text{COP}}_{\text{Right},x}=\frac{\sum_{i=1}^{N}F_{\text{Right},i}\times x_{i}}{\sum_{i=1}^{N}F_{\text{Right},i}},\;{\text{COP}}_{\text{Right},y}=\frac{\sum_{i=1}^{N}F_{\text{Right},i}\times y_{i}}{\sum_{i=1}^{N}F_{\text{Right},i}}$$ (2)

In Equations 1, 2, $F_{\text{Left},i}$ and $F_{\text{Right},i}$ are the forces measured by the i -th sensor on the left and right foot respectively; $x_{i}$ and $y_{i}$ are the horizontal and vertical coordinates of the i -th sensor; and ∑F is the total force applied to the foot.

### 3.4 Classification

In this research, DT, RF, XGBoost, and LightGBM models were applied to the prepared dataset 1 and dataset 2. Further, these models were fine-tuned for accurate diagnosis of PD at various stages. By conducting a set of experiments, the most suitable model for classification was identified. In this research, LightGBM model proved its supremacy (Ko et al., 2022; Li et al., 2025). Now, the LightGBM model is trained on the pre-processed dataset as shown in Equation 3.

$$D=\left\{\left(x_{1},y_{1}\right),\left(x_{2},y_{2}\right),\ldots ,\left(x_{n},y_{n}\right)\right\}$$ (3)

In Equation 3, x represents the input features and y is the corresponding output label. The objective is to find a function F(x) that minimizes the loss. This function is defined in Equation 4.

$$L\left(F\right)=\overset{n}{\underset{i=1}{\sum }}\ell \left(y_{i},F\left(x_{i}\right)\right)$$ (4)

The LightGBM model uses Gradient-based One-Side Sampling (GOSS). It focuses on data points with larger gradients. This helps the model learn better from hard-to-fit examples without being slowed down by easy ones. It also leverages Exclusive Feature Bundling (EFB), which bundles mutually exclusive features to decrease dimensionality and increase efficiency.

Features in LightGBM model can be represented in terms of a leaf nodes of a tree. The tree grows depthwise by adding new leaf nodes with the maximum variance gain. It leads to deeper trees with lower values of loss. This helps in fast convergence and improves accuracy of the model. In addition, LightGBM model provides the option to set a maximum depth to avoid overfitting. In binary classification problems, LightGBM model predicts a target variable y∈{0,1} . The model output $\left(F_{m}\left(x\right)\right.$ at the *m*th iteration is passed through a sigmoid function using Equation 5.

$$\sigma \left(F_{m}\left(x\right)\right)=\frac{1}{1+e^{-F_{m}\left(x\right)}}$$ (5)

This converts the real-valued prediction to a probability in the range 0 through 1 for binary decision-making. The model is trained iteratively. At each iteration i , LightGBM fits a weak learner $h_{i}\left(x\right)$ to the negative gradient (pseudo-residuals) of the loss function and updates the model as shown in Equation 6.

$$F_{i}\left(x\right)=F_{i-1}\left(x\right)+\gamma_{i}h_{i}\left(x\right)$$ (6)

#### 3.4.1 Model training

In this research, SMOTE technique was used to balance the dataset and improve model training. SMOTE was implemented using the imblearn.over_sampling.SMOTE function with parameters sampling_strategy = ‘auto’, k_neighbors = 5, and random state = 42, ensuring balanced class representation in the training data while maintaining reproducibility. SMOTE was applied only to the training dataset to prevent data leakage into the test set. To validate the synthetic data, we compared the feature-wise distributions of the original and synthetic minority samples using kernel density estimates and the Kolmogorov–Smirnov (KS) test. Most of the features showed no significant difference. The ideal sample size is determined by study challenge, feature count and correlation, variability, model complexity, and effect magnitude. Here, we used 50 samples for each feature. To train ML-models on the prepared datasets, Google Colab was used. It provides a runtime access environment integrated with a T4 GPU. Optuna, a hyperparameter optimization package, was used to find the combination of parameters over 50 epochs. Hyperparameters were determined based on the hit and trial experiments on the dataset1 and dataset2. Based on the experiments, number of leaves was set 143, the maximum depth 19, and a learning rate to 0.2516. Furthermore, the number of estimators was optimized to 923, the minimum number of child samples was set to 50, the subsample ratio to 0.8745, and the column sample by tree to 0.8674.

## 4 Performance metrics

The performance of the ML models applied in this research was evaluated in terms of accuracy, precision, recall, and F1-score defined in Equations 7–10 respectively. Further, confusion matrices and receiver operating characteristic (ROC) curves were also produced.

$$\text{Accuracy}=\frac{TP+TN}{TP+TN+FP+FN}$$ (7)

$$\text{Precision}=\frac{TP}{TP+FP}$$ (8)

$$\text{Recall}=\frac{TP}{TP+FN}$$ (9)

$$\text{F}1-\text{Score}=2\cdot \frac{\text{Precision}\cdot \text{Recall}}{\text{Precision}+\text{Recall}}=\frac{2TP}{2TP+FP+FN}$$ (10)

In these equations, TP is the true positive, TN is the true negative, FP is the false positive, and FN is the false negative.

## 5 Results

This section presents the results obtained by applying ML algorithms namely Decision Tree, Random Forest, XGBoost, and LightGBM Models on dataset 1 and dataset 2. The performance of these models is presented in terms of accuracy, precision, recall, F1-score, confusion matrix, and Receiver Operating characteristic (ROC) Curve. Further, the H and Y stages (Stage 0 to Stage 3) of patients obtained by analyzing gait data are also demonstrated.

### 5.1 Performance analysis of classifiers for PD severity assessment on dataset 1

Table 3 presents class-wise results of the above-mentioned classifiers. The results reveal higher efficacy of the LightGBM model than other 3 ML models applied on dataset 1. Also, the LightGBM model exhibits reduced variance across iterations and give reliable outcomes. As shown in Table 3, DT reported a mean accuracy of 90.5% in PD severity assessment. In comparison, RF classifier achieved a mean accuracy of 93.5%, demonstrating superior performance due to ensemble learning over multiple decision trees. Furthermore, XGBoost yielded a mean accuracy of 94.7%, benefiting from gradient boosting and regularization techniques. The LightGBM model reported the highest mean accuracy of 98.2%.

**TABLE 3:** Stage wise performance comparison of classifiers on dataset 1.

Model	Stage	Accuracy	Precision	Recall	F1 score
Decision tree	Stage 0	0.89	0.89	0.89	0.89
	Stage 2	0.88	0.91	0.88	0.90
	Stage 2.5	0.92	0.93	0.92	0.92
	Stage 3	0.93	0.79	0.93	0.86
	Average	0.905	0.88	0.905	0.8925
Random forest	Stage 0	0.91	0.94	0.91	0.92
	Stage 2	0.91	0.95	0.91	0.93
	Stage 2.5	0.94	0.95	0.94	0.95
	Stage 3	0.98	0.75	0.98	0.85
	Average	0.935	0.8975	0.935	0.9125
XGBoost	Stage 0	0.93	0.94	0.93	0.94
	Stage 2	0.93	0.95	0.93	0.94
	Stage 2.5	0.95	0.96	0.95	0.96
	Stage 3	0.98	0.85	0.98	0.91
	Average	0.9475	0.925	0.9475	0.9375
LightGBM (proposed)	Stage 0	0.98	0.99	0.98	0.98
	Stage 2	0.98	0.98	0.98	0.98
	Stage 2.5	1.00	1.00	1.00	1.00
	Stage 3	0.97	0.96	0.97	0.96
	Average	0.9825	0.9835	0.9825	0.98

The fold-wise performance of the proposed LightGBM model on dataset 1 is summarized in Table 4. The LightGBM model gave the accuracy and precision of 98.91% and recall and F1-score of 98.90%. Stratified 5-fold cross-validation was employed to ensure balanced representation of each class across all folds. The minimal variation between 0.2 and 0.4 among the five-fold results confirms reliability of the LightGBM model.

**TABLE 4:** Fold-wise performance of the LightGBM model on dataset 1.

Fold	Accuracy	Precision	Recall	F1 score
Fold 1	0.9894	0.9895	0.9866	0.9881
Fold 2	0.989	0.9889	0.98889	0.9889
Fold 3	0.989	0.9889	0.9889	0.9889
Fold 4	0.989	0.989	0.989	0.989
Fold 5	0.9891	0.9891	0.9891	0.9891
Average	0.9891	0.9891	0.989	0.989

**FIGURE 3:**
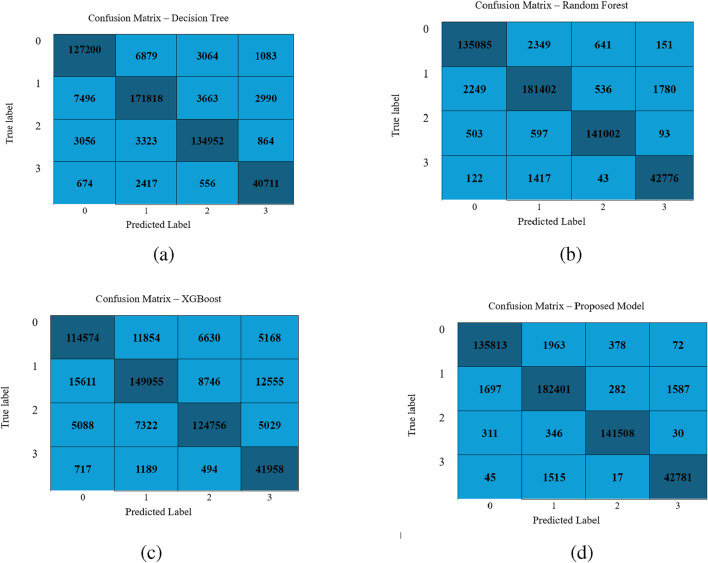
Performance visualization of different classifiers: **(a)** Decision Tree, **(b)** Random Forest, **(c)** XGBoost, and **(d)** LightGBM Model.

FIGURE 3 illustrates the confusion matrix for the classifiers Decision Tree, Random Forest, XGBoost, and LightGBM. The confusion matrix reveals that the LightGBM model shows minimal misclassifications and proves its supremacy.

**FIGURE 4:**
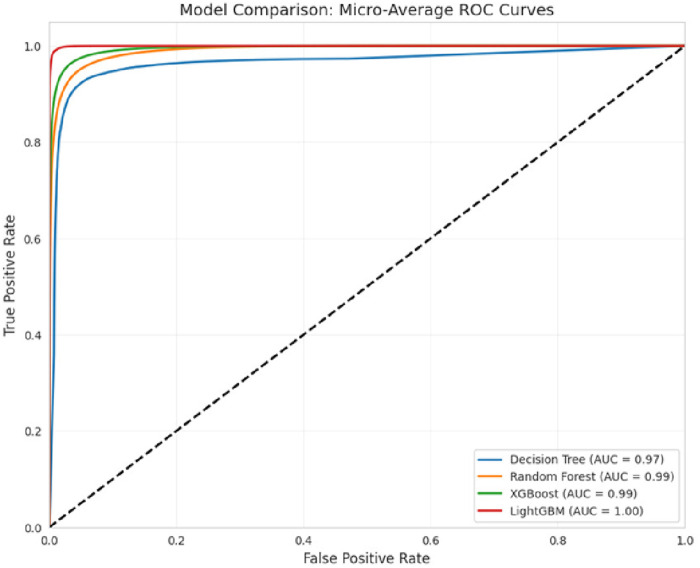
ROC curve of the classifiers.

To assess the performance of the LightGBM model, a Receiver Operating Characteristic (ROC) curve was generated for the multiclass classification task. As shown in Figure 4, the ROC curve illustrates the trade-off between true positive rate and false positive rate across various classification thresholds. The results demonstrate that the model is an effective classifier for all classes, with area under the curve (AUC) values approaching 1. This indicates the model’s high capability to accurately differentiate among the 4 H& Y stages with strong confidence.

### 5.2 Performance analysis of classifiers for PD severity assessment on dataset 2

Table 5 presents the class-wise performance of Decision Tree, Random Forest, XGBoost, and LightGBM classifiers. The results reveal the supremacy of LightGBM model as compared to other models.

**TABLE 5:** Stage wise performance comparison of classifiers on dataset 2.

Model	Stage	Accuracy	Precision	Recall	F1 score
Decision tree	Stage 1	0.73	0.92	0.73	0.81
	Stage 2	0.99	0.87	0.99	0.92
	Stage 3	0.76	0.97	0.76	0.85
	Average	0.82	0.92	0.83	0.86
Random forest	Stage 1	0.63	1.00	0.69	0.78
	Stage 2	1.00	0.85	1.00	0.92
	Stage 3	0.76	0.99	0.76	0.86
	Average	0.79	0.95	0.82	0.85
XGBoost	Stage 1	0.90	0.22	0.90	0.35
	Stage 2	0.64	0.92	0.65	0.76
	Stage 3	0.68	0.84	0.69	0.76
	Average	0.74	0.66	0.74	0.62
LightGBM	Stage 1	0.80	1.00	0.80	0.89
	Stage 2	1.00	0.86	1.00	0.93
	Stage 3	0.76	0.99	0.76	0.86
	Average	0.85	0.95	0.85	0.89

The fold-wise performance of the LightGBM model on dataset 2 is summarized in Table 6.

**TABLE 6:** Fold-wise performance of the LightGBM model on dataset 2.

Fold	Accuracy	Precision	Recall	F1 score
Fold 1	0.8889	0.93	0.80	0.85
Fold 2	0.8911	0.94	0.80	0.85
Fold 3	0.8713	0.93	0.78	0.83
Fold 4	0.8861	0.94	0.79	0.85
Fold 5	0.8812	0.93	0.77	0.83
Average	0.8837	0.93	0.78	0.84

In this study, 5-fold cross-validation along with independent test evaluation was performed to assess the generalizability of the proposed model. The evaluations were conducted on two datasets: dataset-1 and dataset-2. Using the LightGBM classifier, a mean training accuracy of 0.9891 was achieved across the five folds on dataset 1, while the independent test set yielded an accuracy of 0.8837 on dataset 2. These results demonstrate the robustness and generalizability of the proposed approach. The results are summarized in Table 4, 6.

**FIGURE 5:**
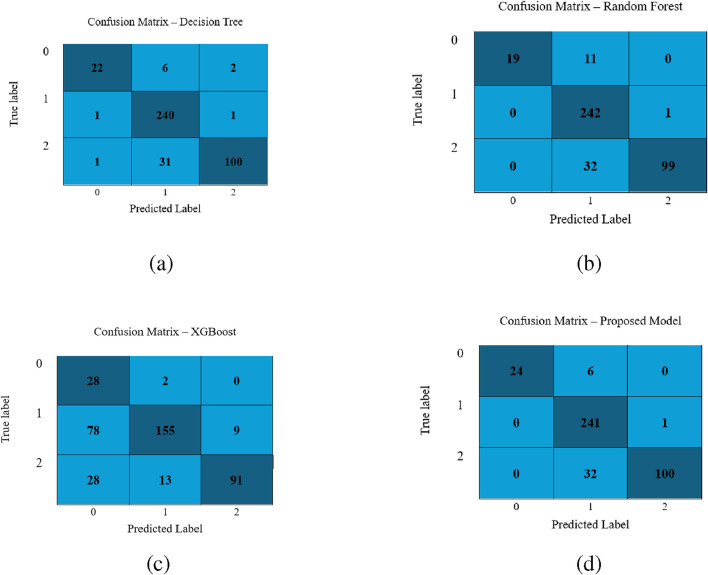
Performance visualization of different classifiers for the second dataset: **(a)** Decision Tree, **(b)** Random Forest, **(c)** XGBoost, and **(d)** LightGBM model.

Further, the confusion matrices obtained by applying DT, RF, XGBoost and LightGBM models using dataset 2 are demonstrated in Figure 5. It is evident from the confusion metrics that all the above-mentined classifiers report the minimum number of misclassifications, especially for class 1, which has the largest sample sizes. However, the LGBM model outperforms the other three classifiers. It correctly classifies 24/30 samples of class 0, 241/242 of class 1, and 100/132 of 2. It makes less number of misclassifications. These findings demonstrate that each model is effective, and LGBM is better than the other three models, specifically for class 0 and 2.

**FIGURE 6:**
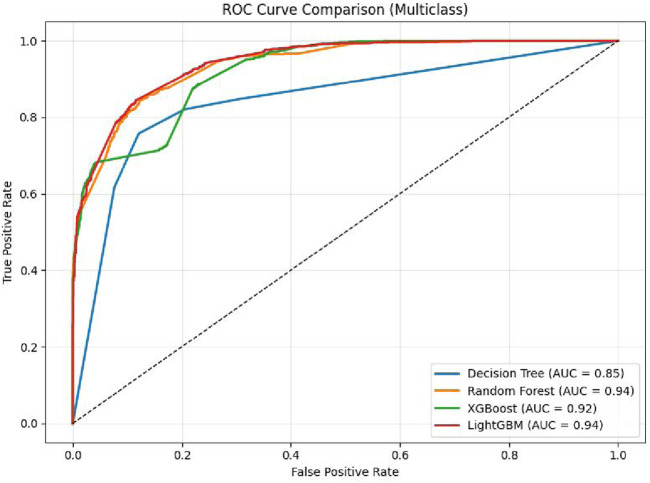
ROC curve of Classifiers using dataset 2.

The comparison plot of the ROC curve obtained by applying DT, RF, XGBoost, and Light GBM models on dataaset 2 is shown in Figure 6. It is evident from the curve that the AUC of the LightGBM Model is 0.9617. It is higher than 0.9555 and 0.9410 values reported by the RF and DT respectively. Moreover, it maintains a balance between precision and recall. It is also apparent that the curves of all the models are centered towards the top-left position, indicating high values of true and low values of false positive rates.

### 5.3 Performance evaluation on two medication states

The experiments were conducted on the datsset including two medication conditions “ON”, and “OFF”. The foldwise results obtained by applying the LightGBM model on “ON”, and “OFF” states are illustrated in Tables 7, 8 respectively. The model achieved an average accuracy of 0.7436, precision of 0.74, recall of 0.80, and F1-score of 0.70 for the ON state. Similarly, the average accuracy of 0.7496, the precision of 0.73, the recall of 0.82, and the F1 score of 0.68 were reported for the OFF state. These findings indicate that the model maintains comparable performance across both medication states. However, recall is slightly better for the OFF state and F1-score is marginally better for the ON state. This reflects a stable predictive behavior of the model despite pharmacological variations.

**TABLE 7:** Fold-wise performance of LightGBM Model on Dataset 2 with Medication ON.

Fold	Accuracy	Precision	Recall	F1 score
Fold 1	0.7426	0.73	0.80	0.70
Fold 2	0.7277	0.73	0.80	0.69
Fold 3	0.7599	0.75	0.82	0.72
Fold 4	0.7351	0.74	0.80	0.70
Fold 5	0.7525	0.75	0.80	0.71
Average	0.7436	0.74	0.80	0.70

**TABLE 8:** Fold-wise performance of LightGBM Model on Dataset 2 with Medication OFF.

Fold	Accuracy	Precision	Recall	F1 score
Fold 1	0.7553	0.73	0.82	0.69
Fold 2	0.7438	0.72	0.81	0.68
Fold 3	0.7599	0.73	0.82	0.69
Fold 4	0.7537	0.74	0.83	0.69
Fold 5	0.7351	0.72	0.80	0.67
Average	0.7496	0.73	0.82	0.68

### 5.4 Explainable AI

**FIGURE 7:**
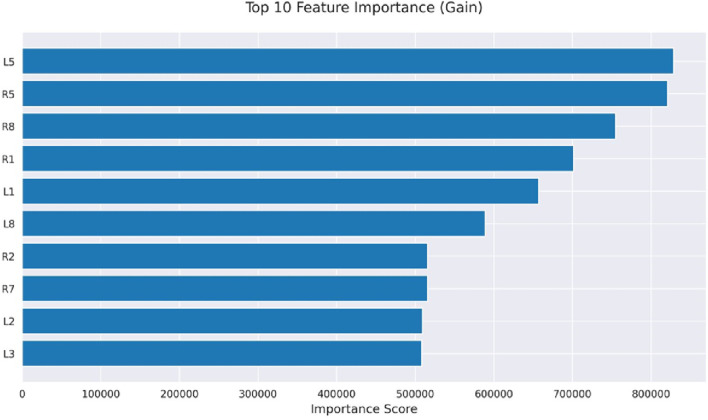
Feature importance.

Initially, machine learning models use gait-based dataset to predict motor impairments in Parkinson’s disease patients. Further, we apply SHAP analysis and explainable AI to provide clinically interpretable insights into the underlying pathophysiology. SHAP values reveal which gait features contribute most to the predictions, including reduced gait speed, increased stride variability, and dual-task performance deficits. By translating these model predictions into feature-specific explanations, explainable AI bridges the gap between computational results and clinical understanding. It also supports early detection, risk stratification, and targeted interventions for patients. In the present study, Explainable AI methods were employed to visualize individual classification pathways generated by the LightGBM classifier for H& Y staging in Parkinson’s disease. The H& Y scale is a well-established clinical measure that grades disease severity from 0 (no symptoms) to 5 (severe disability), encompassing progressive stages of motor impairment. In our study, the sensors (L1, L2, L3, L5, L8), and (R1, R2, R5, R7, R8) are mounted on the left and right legs respectively. The features extracted from these sensors have varied importance as demonstrated in Figure 7. Using visualized decision trees as well as clinical classification models, the goal was to make models more interpretable across their practice and make AI-based diagnostic tools more certain.

#### 5.4.1 Role of XAI in interpreting healthy and diseased patients

**FIGURE 8:**
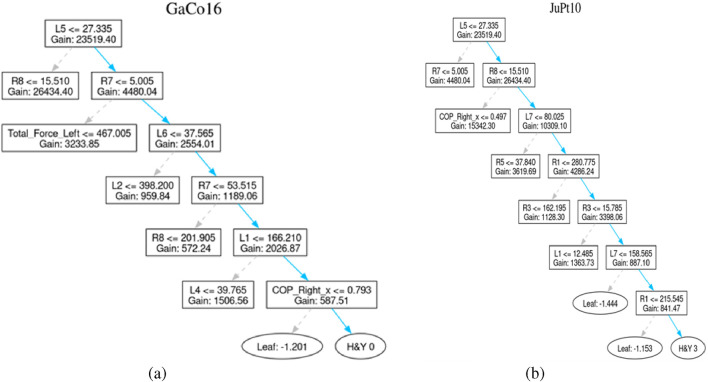
Healthy patient and diseased patient. **(a)** Control subject GaCo16’s tree **(b)** Patient JuPt10’s tree.

The decision tree presented in Figure 8 represents the personal classification trajectories produced by the LightGBM model. This reveals the logical route behind each classification decision. Thus, it allows the clinicians to check questions and interpret the results of the model. This helps in making informed clinical decisions. For example, the path of GaCo16 patient concludes with the H&Y 0 classification. It describes the lack of clinical symptoms of the Parkinson Disease. To draw this conclusion, the model uses a few decision nodes only. This indicates that sound predictive indicators exist in the initial stages of the tree structure.

Similarly, The path of the JuPt10 patient consists of seven decision nodes until it reaches a level of H&Y 3, signifying moderate bilateral disease with part of postural unsteadiness. Complex pattern and subtle progression of the symptoms result in the length of such a path, which can be well explained by the combined features.

The trees generated for both of above-stated patients are shown in Figure 8.

For better understanding, the interpretation of another set of patients classified with H&Y of 2, 2.5, or 3 is also discussed.

For example, Patient SiPt35 is classified with an H&Y stage-1, indicating unilateral involvement with minimal or no functional impairment. The decision path in this case is relatively short, suggesting the classifier quickly identified hallmark signs of early-stage of Parkinson disease.

**FIGURE 9:**
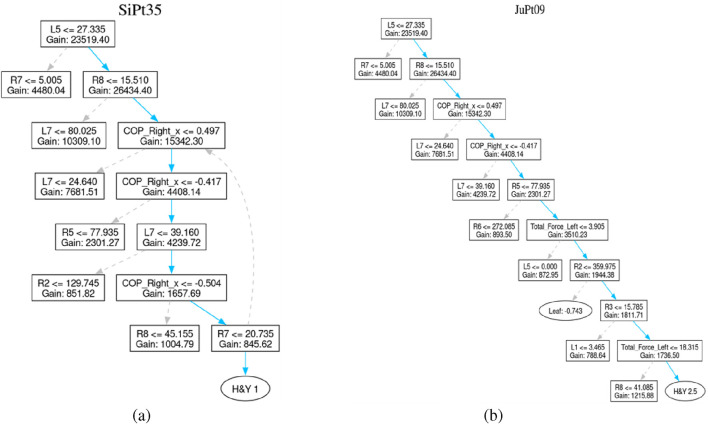
Patient affected by Parkinson’s Disease. **(a)** Patient SiPt35’s tree **(b)** Patient JuPt09’s tree.

Similarly, decision path of Patient JuPt09 moves through 9 decision nodes, culminating in an H&Y 2.5 classification. This stage corresponds to mild bilateral disease with recovery on the pull test. This might involve more nuanced features, explaining the longer decision sequence. The trees generated for these patients are shown in Figure 9.

Such visualization of decision-tree models is a radical step towards the explainability of complex classification models applied for identifying Parkinson Disease staging. These visualizations reveal the signable pathways of every clinical classification. Thus, increases the level of stakeholders trust in AI-based methods. It provides an opportunity to make AI-based individual treatment plans.

### 5.5 Ablation study

To validate the performance of the LightGBM Model, ablation study is performed. The impact of hyperparameters such as learning rate, boosting iterations, and minimum depth was analysed.

Impact of Learning rate: The LightGBM model was trained using different learning rates. The results obtained on 0.5, 0.3, 0.25, and 0.01 learning rates are illustrated in Table 9. However, the boosting iterations were fixed to 923 and the maximum depth to 19 during these experiments. It is apparent from the results that the model reports the highest accuracy on the learning rate of 0.25. Thus, this learning rate was selected for further experiments.

**TABLE 9:** Model performance with varying learning rates (boosting iterations = 923, max depth = 19).

Learning rate	Boosting iterations	Max depth	Accuracy (%)
0.5	923	19	65.18
0.3	923	19	98.28
0.25	923	19	98.39
0.01	923	19	87.49

Impact of Number of Boosting Iterations: To determine the impact of number of boosting iterations, the LightGBM model was trained on various iterations. However, the learning rate was set to 0.25, and the maximum Depth to 19. The results obtained using 500, 923, 1,500, and 1700 boosting iterations.are shown in Table 10. It is clear from the results that the model outperforms on the boosting iterations of 923. Thus, 923 boosting iterations were selected for further experiments.

**TABLE 10:** Effect of boosting iterations on accuracy (Learning Rate = 0.25, Max Depth = 19).

Boosting iterations	Learning rate	Max depth	Accuracy (%)
500	0.25	19	92.89
923	0.25	19	98.39
1,500	0.25	19	69.35
1700	0.25	19	66.70

Impact of the Maximum depth: To check the impact of the maximum depth, the experiments were conducted on various depths. The results obtained by training the LightGBM model on 15, 17, 19 and 21 depths are demonstrated in Table 11. However, the learning rate was kept as 0.25, and boosting iterations to 923. It is evident from the results that the model reports the highest performance on the depth of 19.

**TABLE 11:** Effect of max depth on model accuracy (Learning Rate = 0.25, Boosting Iterations = 923).

Max depth	Learning rate	Boosting iterations	Accuracy (%)
15	0.25	923	98.22
17	0.25	923	87.73
19	0.25	923	98.39
21	0.25	923	86.93

## 6 Discussion

This study significantly improves the classification accuracy of gait patterns between healthy individuals and PD patients by integrating COP features with LightGBM algorithm. It also addresses the problem of class imbalance using SMOTE technique. The proposed methodology classifies PD severity across H& Y stages, achieving the highest classification accuracy of 98.25% for dataset 1 and 85% for dataset 2.

The accuracy is higher than related works discussed in the literature. For instance, Balakrishnan et al. (2024) reported 94.14% accuracy using an IFS-DT, and Veeraragavan et al. (2020) achieved 97.4% using a neural network model with SMOTE-based cross-validation. Similarly, El Maachi et al. (2020) attained 98.7% with a 1D-Convolutional Neural Network, and Alharthi et al. (2020) reported 95.5% using a Parallel 2D Deep CNN. Another study by Huo et al. (2025) reported 90.8% for 1D-ConvNet and one-dimensional Transformer. The researchers Abdulhay et al. (2018) and Alam et al. (2017), applied SVM models, and reported accuracies of 92.7% and 95.7%, respectively. Similarly, Wu and Krishnan (2009) used a Least Square SVM with a polynomial kernel and achieved 90.32% accuracy. Khoury et al. (2019) reported a range of 83.3%–92.86% using various models. Balaji et al. (2020) reported 99.4% accuracy with DT, Wang et al. (2024) achieved 98.8% using SVM, Aşuroğlu et al. (2018) reached 99% using a Locally Weighted Random Forest (LWRF), and Balaji et al. (2021a) reported up to 98.4% with SVM.

Table 12 shows the comparative performance of different methodologies on dataset 1.

**TABLE 12:** Performance comparison of different methodologies on dataset 1.

References	Year of publication	Methodology	Accuracy (%)
Balakrishnan et al. (2024)	2024	Information set-based DT (IFS-DT)	94.14
Veeraragavan et al. (2020)	2020	Neural network model (cross validation SMOTE)	97.4
El Maachi et al. (2020)	2020	1D-ConvNet	98.7
Alharthi et al. (2020)	2021	Parallel 2D-DCNN	95.5
Huo et al. (2025)	2025	1D-ConvNet and one-dimensional transformer	90.8
Abdulhay et al. (2018)	2018	SVM	92.7
Wu and Krishnan (2009)	2009	Least square SVM with polynomial kernel	90.32
Alam et al. (2017)	2017	SVM classifier	95.7
Khoury et al. (2019)	2019	KNN, DT, RF, NB, SVM	83.3–92.86
Balaji et al. (2020)	2020	DT, SVM, EC, BC	DT: 99.4
Wang et al. (2024)	2024	SVM classifier	98.8
Aşuroğlu et al. (2018)	2018	LWRF	99
Balaji et al. (2021a)	2021	KNN, EC, NB, SVM	SVM: 98.4
Proposed work		LightGBM	98.25

Table 13 shows the comparison of existing methodologies applied on dataset 2.

**TABLE 13:** Comparison of Existing Models applied on dataset 2.

References	Year of publication	Methodology	Results
Xu et al. (2025)	2025	Graph-based multimodal fusion framework (GMFF) using GAT + GCCA + transformer + double-hurdle module	Accuracy (FOG detection): 0.978
Lochhead and Fisher (2025)	2025	1-Stream ST-GCN with attention on temporal dimension	Accuracy: 94.4%
Adeli et al. (2024)	2024	6 motion encoder models (best: PoseFormerV2) + feature-based baseline	62%
Gonçalves et al. (2025)	2025	LSTM	Accuracy: 91.7
Proposed work		LightGBM	85

For example, Xu et al. (2025) demonstrates the Graph-based Multimodal Fusion Framework (GMFF) that can include kinematic, kinetic, and spatiotemporal gait characteristics to determine the severity of Freezing of Gait (FoG) in Parkinson disease. Lochhead and Fisher (2025) presents a low-computation ST-GCN model with a new synthetic dataset (WeightGait) for detecting gait abnormalities under at-home conditions. Adeli et al. (2024) uses six modern motion encoders to estimate severity ratings of Parkinson Disease using gait and demonstrates the possible viability of optimized models like PoseFormerV2 in the clinical practice. Gonçalves et al. (2025) develop three distinct LSTM-based models using inertial sensor data from a custom cohort of 40 Parkinson’s disease patients to classify (1) disease stage (H), (2) motor impairment, and (3) quality of life, achieving accuracies of 89%, 91.7%, and 87.8%, respectively. It is apparent from the results obtained that the state-of-the-art approaches lack in handling the class imbalance. It can lead to reduced sensitivity for H and Y stages. The approach proposed in this research addresses this issue through SMOTE. It enhances the performance across all PD stages. The dataset used in this research includes patients at varying stages of PD, measured via the H and Y scale. The experiments confirm that early-stage PD patients (H& Y stages 1–2) can be correctly identified. This suggests the model’s potential for early diagnosis.

### 6.1 Insights on cognitive decline

Studies in literature show that gait-based ML models may not only capture motor impairments but could also serve as early indicators of cognitive decline risk. It reflects subtle neural and functional changes that precede clinically detectable symptoms (Kang et al., 2022). Further, it is highlighted that gait alterations such as reduced gait speed, increased stride time variability, and impaired balance precede measurable cognitive deficits, suggesting that these gait features could serve as early indicators of cognitive decline. Slower gait speed and increased gait variability lead to mild cognitive impairment (MCI) and dementia progression (Verghese et al., 2007; Beauchet et al., 2016). Similarly, a strong interrelation between gait and cognition in Parkinson’s disease is reported in Iansek et al. (2013). Thus, deficits in executive control and attention are reflected in gait disturbances like freezing, irregular cadence, and difficulty with dual-task walking. These may be signals for early cognitive dysfunction. Complementing these findings, movement disorders demonstrated that neural hyperactivity within motor and cerebellar circuits contributes to both motor and cognitive impairments (Yu et al., 2007). Similarly, dual-task walking performance reveals cognitive load sensitivity even in individuals without clinical symptoms (Montero-Odasso et al., 2012). This indicates that gait irregularities may mirror early changes in brain regions implicated in cognitive processing. Therefore, these changes should be interpreted cautiously in correlation with the influence of age, neurological conditions, and comorbidities.

## 7 Conclusion

This study successfully developed a fine-tuned LightGBM model for the classification of Parkinson’s Disease patients based on the disease severity using Gait dataset. The study also presents the performance comparison of Decision Tree, Random Forest, XGBoost, and Light GBM models. The comparison proves the supremacy of the fine-tuned LightGBM model on dataset 1 and dataset 2. The model reported the highest accuracy of 98.25%. Furthermore, the study employed the explainabile AI to illstrate the decision making mechanism of the LightGBM model. This improves the trust of stakeholders in adopting the proposed framework for parkinson disease detection, and classification. This research determined the impact of medications states “ON” and “OFF” on the performance of the model applied for parkinson disease classification. This improves the reliability and practical applicability of the proposed framework. Next, to validate the performance of model and to determine impact of hyperparameters, ablation study was performed. Based on the ablation study, it is concluded that the LightGBM model shows its highest performance of more than 98% on the learning rate of 0.25, boosting iterations of 923, and the maximum depth of 19. Thus, the proposed framework can be used as techno-assistant for clinicians in making more informed decisions about classification, early detection, and severity stage prediction of patients suffering from parkinson disease.

## 8 Limitations and future scope

The study was conducted using publicly available open-access datasets, which may inherently introduce sampling bias due to variations in data collection protocols and participant characteristics. While such datasets enable reproducibility and transparency, they may not fully capture real-world clinical heterogeneity. We also recognize that the current work lacks real-time clinical validation, which is essential to confirm the model’s performance in practical settings. As part of future work, we plan to collaborate with clinical partners to conduct prospective data collection and real-time testing of the model, thereby addressing potential sampling biases and enhancing the clinical applicability and external validity of our findings. Future work will also focus on real-world testing using wearable sensors to capture continuous and naturalistic gait patterns. This can provide more accurate insights into patient mobility in daily life. Furthermore, incorporating larger datasets will enable better tracking of disease progression over time. Finally, the inclusion of external validation cohorts is planned to ensure the generalizability, reliability, and clinical applicability of the proposed model across diverse populations.

## Data Availability

The data utilized in this research were obtained from an open-source dataset. The links are as follows: 1. https://physionet.org/content/gaitpdb/1.0.0/ 2. https://figshare.com/articles/dataset/A_dataset_of_overground_walking_full-body_kinematics_and_kinetics_in_individuals_with_Parkinson_s_disease/14896881.

## References

[B1] Abdulhay E. Arunkumar N. Narasimhan K. Vellaiappan E. Venkatraman V. (2018). Gait and tremor investigation using machine learning techniques for the diagnosis of parkinson disease. Future Gener. Comput. Syst. 83, 366–373. 10.1016/j.future.2018.02.009

[B2] Adeli V. Mehraban S. Ballester I. Zarghami Y. Sabo A. Iaboni A. (2024). Benchmarking skeleton-based motion encoder models for clinical applications: estimating parkinson’s disease severity in walking sequences. arXiv [cs.CV], 1–10. 10.1109/fg59268.2024.10581933

[B3] Afzal N. Ramzan B. Fatima N. Khalid M. Waris A. Gilani S. O. (2023). “Plantar pressure response for classification of parkinson’s disease patients with gait abnormalities using machine learning,” in 2023 3rd international conference on digital futures and transformative technologies (ICoDT2) (IEEE), 1–6.

[B4] Agarwal M. Rani G. Kumar A. K P. K. Manikandan R. Gandomi A. H. (2024). Deep learning for enhanced brain tumor detection and classification. Results Eng. 22, 102117. 10.1016/j.rineng.2024.102117

[B5] Alam M. N. Garg A. Munia T. T. K. Fazel-Rezai R. Tavakolian K. (2017). Vertical ground reaction force marker for parkinson’s disease. PloS One 12, e0175951. 10.1371/journal.pone.0175951 28493868 PMC5426596

[B6] Alharthi A. S. Casson A. J. Ozanyan K. B. (2020). Gait spatiotemporal signal analysis for parkinson’s disease detection and severity rating. IEEE Sensors J. 21, 1838–1848. 10.1109/jsen.2020.3018262

[B7] Aşuroğlu T. Açıcı K. Erdaş Ç. B. Toprak M. K. Erdem H. Oğul H. (2018). Parkinson’s disease monitoring from gait analysis *via* foot-worn sensors. Biocybern. Biomed. Eng. 38, 760–772. 10.1016/j.bbe.2018.06.002

[B8] Balaji E. Brindha D. Balakrishnan R. (2020). Supervised machine learning based gait classification system for early detection and stage classification of parkinson’s disease. Appl. Soft Comput. 94, 106494. 10.1016/j.asoc.2020.106494

[B9] Balaji E. Brindha D. Umesh K. K U. (2021a). Data-driven gait analysis for diagnosis and severity rating of parkinson’s disease. Med. Eng. and Phys. 91, 54–64. 10.1016/j.medengphy.2021.03.005 34074466

[B10] Balaji E. Brindha D. Vikrama R. (2021b). Automatic and non-invasive parkinson’s disease diagnosis and severity rating using lstm network. Appl. Soft Comput. 108, 107463. 10.1016/j.asoc.2021.107463

[B11] Balakrishnan A. Medikonda J. Pramod K. Manikandan N. (2024). Information set based decision tree for parkinson’s disease severity assessment using multidimensional gait dataset. IEEE Access 12, 129187–129201. 10.1109/access.2024.3456438

[B12] Beauchet O. Allali G. Sekhon H. Verghese J. Guilain S. Steinmetz J. P. (2016). Motoric cognitive risk syndrome: could it be defined through increased gait variability? Findings from the “gait and alzheimer interactions tracking” study. Front. Aging Neurosci. 8, 172. 10.3389/fnagi.2018.00434 27458376

[B13] Boari D. (2022). A dataset of overground walking full-body kinematics and kinetics in individuals with parkinson’s disease 10.3389/fnins.2023.992585 PMC9978741 36875659

[B14] Brand Y. E. Schwartz D. Gazit E. Buchman A. S. Gilad-Bachrach R. Hausdorff J. M. (2022). Gait detection from a wrist-worn sensor using machine learning methods: a daily living study in older adults and people with parkinson’s disease. Sensors 22, 7094. 10.3390/s22187094 36146441 PMC9502704

[B15] El Maachi I. Bilodeau G.-A. Bouachir W. (2020). Deep 1d-convnet for accurate parkinson disease detection and severity prediction from gait. Expert Syst. Appl. 143, 113075. 10.1016/j.eswa.2019.113075

[B16] Faiem N. Asuroglu T. Acici K. Kallonen A. Van Gils M. (2024). “Assessment of parkinson’s disease severity using gait data: a deep learning-based multimodal approach,” in Nordic conference on digital health and wireless solutions (Springer), 29–48.

[B17] Gonçalves H. R. Pinheiro P. Pinheiro C. Martins L. Rodrigues A. M. Santos C. P. (2025). Deep learning models for improving parkinson’s disease management regarding disease stage, motor disability and quality of life. Comput. Biol. Med. 189, 109961. 10.1016/j.compbiomed.2025.109961 40037167

[B18] Hausdorff J. M. (2008). Gait in parkinson’s disease

[B19] Huo H. Jiao S. Li D. Ma L. Xu N. (2025). Efficient quantification of parkinson’s disease severity using augmented time-series data. PloS One 20, e0319826. 10.1371/journal.pone.0319826 40173391 PMC11964457

[B20] Iansek R. Huxham F. McGinley J. (2013). Gait and cognition in Parkinson’s disease: implications for rehabilitation. Rev. Neurosci. 24, 293–300. 10.1515/revneuro-2013-0006 23645123

[B21] Jankovic J. (2008). Parkinson’s disease: clinical features and diagnosis. J. Neurology, Neurosurgery and Psychiatry 79, 368–376. 10.1136/jnnp.2007.131045 18344392

[B22] Kang H. G. Kim J. Lee J. Koh S. B. (2022). Mild cognitive impairment is associated with poor gait performance in patients with Parkinson’s disease. Front. Aging Neurosci. 14, 1003595. 10.3389/fnagi.2022.1003595 36268193 PMC9577227

[B23] Khoury N. Attal F. Amirat Y. Oukhellou L. Mohammed S. (2019). Data-driven based approach to aid parkinson’s disease diagnosis. Sensors 19, 242. 10.3390/s19020242 30634600 PMC6359125

[B24] Ko P.-C. Lin P.-C. Do H.-T. Huang Y.-F. (2022). P2p lending default prediction based on ai and statistical models. Entropy 24, 801. 10.3390/e24060801 35741522 PMC9222552

[B25] Kundu N. Rani G. Dhaka V. S. (2020). “Machine learning and iot based disease predictor and alert generator system,” in 2020 fourth international conference on computing methodologies and communication (ICCMC), 764–769. 10.1109/ICCMC48092.2020.ICCMC-000142

[B26] Li L.-H. Sharma A. K. Cheng S.-T. (2025). Explainable ai based lightgbm prediction model to predict default borrower in social lending platform. Intelligent Syst. Appl. 26, 200514. 10.1016/j.iswa.2025.200514

[B27] Lilienfeld D. E. Perl D. P. (1993). Projected neurodegenerative disease mortality in the United States, 1990–2040. Neuroepidemiology 12, 219–228. 10.1159/000110320 8272181

[B28] Lochhead C. Fisher R. B. (2025). A lightweight approach to gait abnormality detection for at home health monitoring. Comput. Biol. Med. 190, 110076. 10.1016/j.compbiomed.2025.110076 40164030

[B29] Marin-Lopez A. Martinez-Martinez F. Martínez-Cadena J. Alvarez-Ramirez J. (2024). Multiscale svd entropy for the analysis of gait dynamics. Biomed. Signal Process. Control 87, 105439. 10.1016/j.bspc.2023.105439

[B30] Marsico M. D. Mecca A. (2019). A survey on gait recognition *via* wearable sensors. ACM Comput. Surv. (CSUR) 52, 1–39. 10.1145/3340293

[B31] Montero-Odasso M. Verghese J. Beauchet O. Hausdorff J. M. (2012). Gait and cognition: a complementary approach to understanding brain function and the risk of falling. J. Am. Geriatrics Soc. 60, 2127–2136. 10.1111/j.1532-5415.2012.04209.x 23110433 PMC3498517

[B32] Mujib M. D. Rao A. Z. Haque M. F. U. Alokaily A. O. Hussain S. S. Aldohbayb A. A. (2025). Modulated theta band frequency with binaural beat stimulation correlates with improved cognitive scores in alzheimer’s patients. Front. Aging Neurosci. 17, 1543282. 10.3389/fnagi.2025.1543282 40099247 PMC11911351

[B33] Pradhan N. Dhaka V. S. Rani G. Chaudhary H. (2022). Machine learning model for multi-view visualization of medical images. Comput. J. 65, 805–817. 10.1093/comjnl/bxaa111

[B34] Raghuvanshi A. Mitra P. Rane D. Kumar S. D. Lahiri U. (2024). Gait wear: an augmented wearable system for gait quantification. IEEE Sensors J. 24, 35673–35685. 10.1109/jsen.2024.3456907

[B35] Senturk Z. K. (2020). Early diagnosis of parkinson’s disease using machine learning algorithms. Med. Hypotheses 138, 109603. 10.1016/j.mehy.2020.109603 32028195

[B36] Veeraragavan S. Gopalai A. A. Gouwanda D. Ahmad S. A. (2020). Parkinson’s disease diagnosis and severity assessment using ground reaction forces and neural networks. Front. Physiology 11, 587057. 10.3389/fphys.2020.587057 33240106 PMC7680965

[B37] Verghese J. Lipton R. B. Hall C. B. Kuslansky G. Katz M. J. Buschke H. (2007). Abnormality of gait as a predictor of non-alzheimer’s dementia. N. Engl. J. Med. 357, 1422–1430. 10.1056/NEJMoa020441 12456852

[B38] Wang Q. Zeng W. Dai X. (2024). Gait classification for early detection and severity rating of parkinson’s disease based on hybrid signal processing and machine learning methods. Cogn. Neurodynamics 18, 109–132. 10.1007/s11571-022-09925-9 38406205 PMC10881932

[B39] Wu Y. Krishnan S. (2009). Statistical analysis of gait rhythm in patients with parkinson’s disease. IEEE Trans. Neural Syst. Rehabilitation Eng. 18, 150–158. 10.1109/tnsre.2009.2033062 20650700

[B40] Xu N. Wang C. Peng L. Zhou X.-H. Chen J. Cheng Z. (2025). A graph-based multimodal fusion framework for assessment of freezing of gait in parkinson’s disease. IEEE Trans. Neural Syst. Rehabilitation Eng. 33, 1539–1549. 10.1109/tnsre.2025.3561942 40244843

[B41] Yu H. Sternad D. Corcos D. M. Vaillancourt D. E. (2007). Role of hyperactive cerebellum and motor cortex in parkinson’s disease. Mov. Disord. 25, 1034–1039. 10.1016/j.neuroimage.2006.11.047 17223579 PMC1853309

[B42] Zeng W. Liu F. Wang Q. Wang Y. Ma L. Zhang Y. (2016). Parkinson’s disease classification using gait analysis *via* deterministic learning. Neurosci. Letters 633, 268–278. 10.1016/j.neulet.2016.09.043 27693437

[B43] Zhao H. Wang R. Lei Y. Liao W.-H. Cao H. Cao J. (2022). Severity level diagnosis of parkinson’s disease by ensemble k-nearest neighbor under imbalanced data. Expert Syst. Appl. 189, 116113. 10.1016/j.eswa.2021.116113

